# Optimizing efficiency in CT services: a data-driven approach using action logs from the radiology information system

**DOI:** 10.1007/s11604-026-02034-1

**Published:** 2026-06-15

**Authors:** Shingo Kayano, Hideki Ota, Yoshihiko Takubo, Hiromu Mashiko, Kosuke Usui, Kazuki Shimada, Minori Hoshika-Kayano, Kei Takase

**Affiliations:** 1 Department of Radiological Technology, Tohoku University Hospital, Sendai, Miyagi Japan; 2 Department of Diagnostic Radiology, Tohoku University Hospital, 1-1 Seiryo-Machi Aoba-Ku, Sendai, Miyagi Japan; 3 Medical IT Center, Tohoku University Hospital, Sendai, Miyagi Japan; 4 Graduate School of Management, GLOBIS University, Tokyo, Japan; 5 Department of Development Promotion, Clinical Research Innovation and Education Center, Tohoku University Hospital, Sendai, Miyagi Japan

**Keywords:** Computed tomography, Radiology information system, Workflow, Quality improvement, Efficiency

## Abstract

**Purpose:**

To evaluate whether Radiology Information System (RIS) action log-guided continuous quality improvement (KAIZEN) improved CT operational efficiency.

**Materials and methods:**

This single-center retrospective before-and-after quality improvement study included 150,540 weekday CT examinations from 2018 to 2022. Five workflow interventions were introduced sequentially from January through February 2021. Daily median examination time, daily median patient entry/exit time, and the end time of the final weekday CT examination were analyzed using segmented interrupted time-series regression with interruptions in January 2020 and January 2021 and heteroskedasticity- and autocorrelation-consistent standard errors. Key performance indicator (KPI) models were adjusted for daily volume, day of the week, and inpatient proportion; unadjusted models were used as sensitivity analyses.

**Results:**

Daily median examination time decreased from 351 s at baseline to 328 s after KAIZEN. KAIZEN initiation was associated with an immediate reduction in adjusted examination time (− 17.37 s; 95% CI, − 31.35 to − 3.40 s; *p* = 0.015), whereas the unadjusted sensitivity analysis was directionally consistent but non-significant (− 11.07 s; *p* = 0.060). Entry/exit time decreased from 216 to 198 s and showed a significant adjusted immediate reduction (− 15.99 s; 95% CI, − 23.93 to − 8.04 s; *p* < 0.001), consistent with the unadjusted analysis. The final weekday CT examination ended 12.49 min earlier (95% CI, − 22.11 to − 2.87 min; *p* = 0.011).

**Conclusion:**

RIS action log-guided KAIZEN was temporally associated with shorter turnover, earlier completion of weekday operations, and reduced adjusted examination time. Replication in diverse settings is warranted.

Secondary Abstract.

In this single-center study, RIS action log-guided KAIZEN was temporally associated with shorter CT patient entry/exit time, earlier weekday completion, and reduced adjusted examination time, supporting routine log-based workflow monitoring.

**Supplementary Information:**

The online version contains supplementary material available at https://doi.org/10.1007/s11604-026-02034-1.

## Introduction

In 2020, Japan had approximately 116 computed tomography (CT) scanners per million population, in contrast to the average of 30 CT scanners per million people across member countries of the Organization for Economic Co-operation and Development (OECD) [1]. As of the 2023 report, the number of CT scanners in Japanese medical institutions was reported at 14,595 [2], making Japan the country with the highest number of CT scanners. Moreover, according to the National Database (NDB) of the Japanese Ministry of Health, Labour and Welfare, the number of CT examinations in Japan has increased by 12% between 2014 and 2021 (increased from 28,145,763 to 31,646,225) [3].

Two major factors have been reported as contributors to the increasing number of CT examinations in Japan. First, Japan’s aging population [4, 5] increases demand because higher prevalence of chronic diseases necessitates more frequent diagnostic imaging. Second, technological advances in CT imaging contribute to increased use; newer scanners are faster, more accurate, and enable lower radiation dose, which improves diagnostic precision and reduces patient discomfort and radiation exposure [6–9]. On this basis, demand for CT examinations has been projected to increase, which necessitates improvements in service efficiency.

In this context, the challenges faced by the CT section can be categorized into issues affecting patients, staff, and administration. Lengthy waiting times, both for appointments and on the examination day, lead to patient anxiety, diagnostic delays, and overall dissatisfaction. CT section staff, including radiologists, nurses, and technologists, face unique challenges: high patient volumes and complex procedures during the examination frequently lead to overtime work. This leads to physical and mental exhaustion, potentially degrading quality of work and well-being. On the administrative front, outsourcing radiological services may reduce short-term operational burdens but can simultaneously reduce hospital revenue. These challenges illustrate the need for improved efficiency and resource management in radiology departments to improve patient care, staff well-being, and administrative outcomes [10–12].

To address these issues, we used Radiology Information System (RIS) action logs to identify operational bottlenecks and guide targeted workflow changes. We applied KAIZEN methodology [13–15] (a Japanese term meaning “continuous improvement,” originally developed in manufacturing and widely adopted in healthcare quality improvement) as a framework for continuous improvement and implemented incremental interventions aimed at reducing turnaround times and strengthening routine CT operations. We evaluated operational performance using two KPIs derived from RIS action logs and a day-level KGI reflecting the end time of the final CT examination.

Accordingly, our research question was whether RIS action logs can quantify changes in CT workflow performance during KAIZEN interventions, with particular focus on changes in the end time of daily operations as a key goal indicator.

## Materials and methods

This retrospective study used routinely collected RIS action logs. This work did not constitute human subjects research under the Japanese Ethical Guidelines for Life Science and Medical Research Involving Human Subjects; therefore, institutional review board approval and informed consent were not required.

### Overview of our CT section

Our academic hospital has 1,160 inpatient beds and averages 3,115 outpatient visits per day. The imaging center was equipped with three multi-slice CT scanners: SOMATOM Definition Flash (Siemens Healthineers, Forchheim, Germany), Aquilion Precision, and Aquilion *ONE* ViSION Edition (Canon Medical Systems, Otawara, Japan). The RIS was ACTRIS (J-MAC SYSTEM, Inc., Sapporo, Japan).

**Fig. 1:**
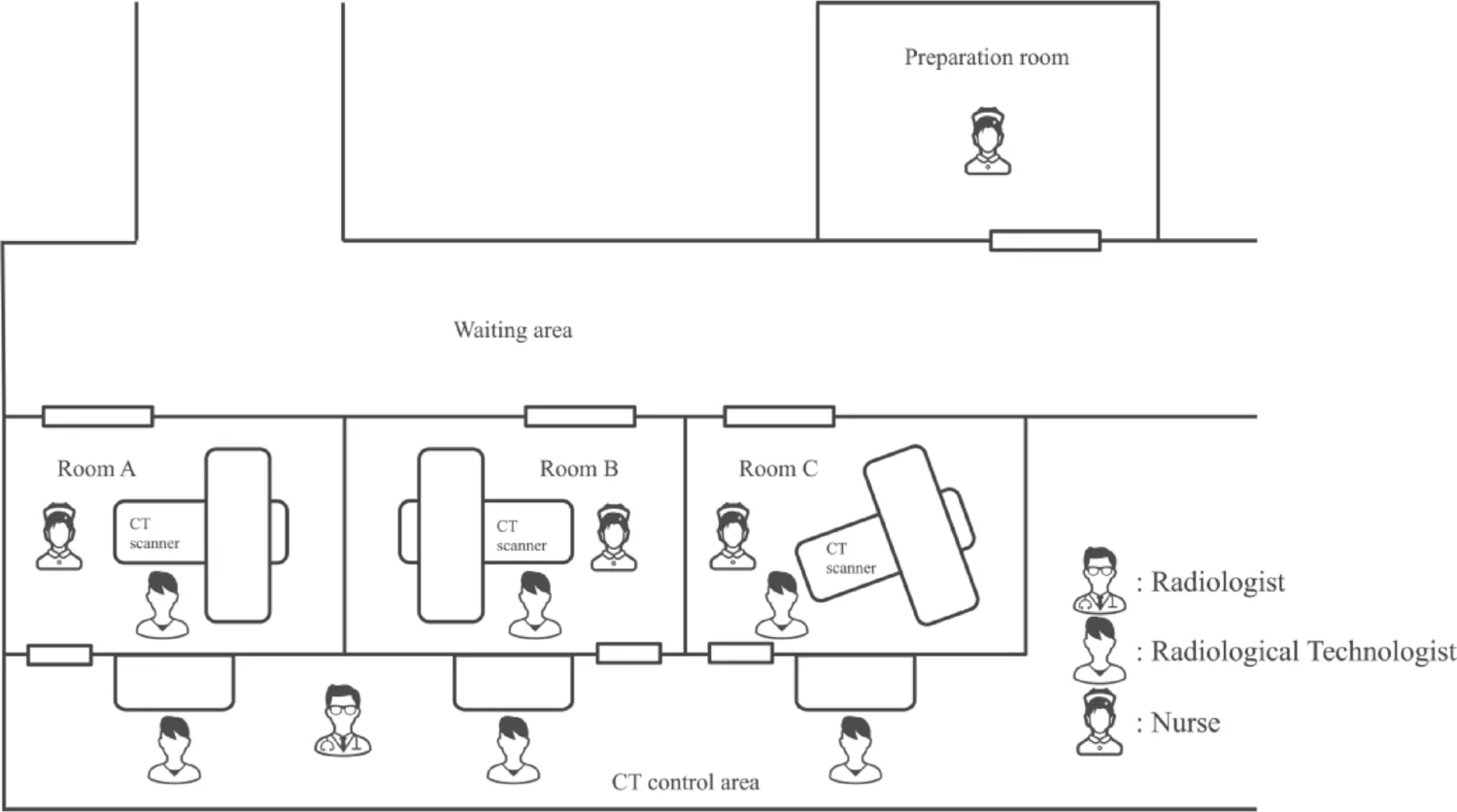
Floor layout of the CT imaging department. The department includes three CT scanning rooms (**A**–**C**). During daytime operations, each room is typically staffed by two radiological technologists and one nurse, and examinations are supervised by a radiologist from the adjacent control area. A preparation room, staffed by an intravenous injection nurse (IV nurse) for contrast-enhanced CT examinations, is located near the central waiting area. The reception desk is not shown in this figure

Regular daytime services operated from 8:30–17:15. Two radiological technologists and one nurse were assigned to each CT scanner. An intravenous injection nurse (IV nurse) was stationed in the preparation room for contrast-enhanced CT examinations. One on-site radiologist supervised examinations, including protocol and image-quality checks, rapid preliminary review of findings, and management of acute adverse reactions to iodinated contrast agents. Figure 1 illustrates the CT section layout and personnel assignments.

A dedicated hybrid emergency room system and an additional CT scanner were installed in a separate area within the Advanced Medical Care Center. Patients in the intensive care unit (ICU), high care unit (HCU), and emergency department were primarily scanned using this separate system. Therefore, the three CT scanners in the imaging center predominantly served outpatients and general ward inpatients, and critically ill or emergency patients were largely excluded from the present workflow analysis. Importantly, patients with suspected or confirmed COVID-19 infection were directed to the dedicated emergency CT system and were not scanned using the imaging center CT scanners throughout the study period. As a result, infection control measures specific to COVID-19 patients (e.g., extended room ventilation intervals, additional personal protective equipment protocols) were not applied in the imaging center workflow and did not directly affect the KPIs analyzed in this study. No replacement of CT scanners or the information system occurred during the study period.

### Quantitative analysis of daily workflow using RIS action logs within a KAIZEN framework

**Fig. 2:**
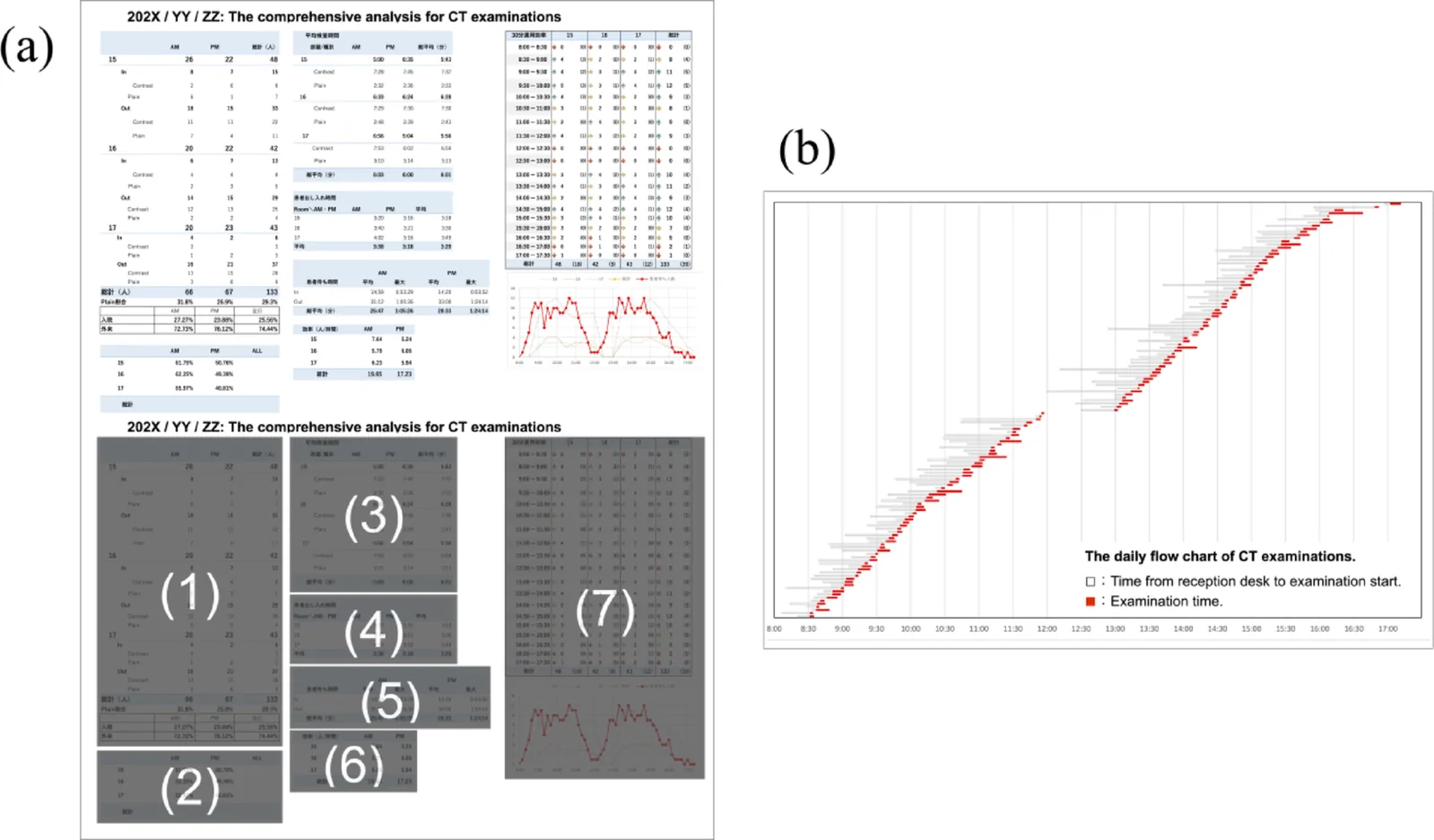
Daily CT examination reports generated from the radiology information system (RIS). **a** The report includes: (1) Examination attributes (contrast/plain proportion, inpatient/outpatient ratio), (2) The rate of operation, (3) Mean examination durations (contrast and plain, overall mean), (4) Mean patient entry/exit times, (5) Patient waiting times (inpatient/outpatient averages, maximums), (6) The number of examinations per hour, and (7) Examination distribution across time slots (8:00–17:30). **b** Daily flow of CT examinations. The horizontal axis represents time (8:00–17:30) in 30-min intervals. Each bar represents an examination, with color indicating stage: □ (white square), reception to examination start; ■ (red square), examination duration. Examinations across all three CT rooms are displayed simultaneously, visualizing parallel processes and room utilization. These reports characterize examination processes, patient flow, and operational efficiency, and inform process optimization and service improvement

As part of a continuous quality improvement (KAIZEN) initiative, RIS action logs were used to visualize daily workflow. After completion of daily operations, action logs were extracted and included the following variables: reception time; inpatient/outpatient status; non-contrast vs. contrast-enhanced examination; CT room; examination start time; and examination end time. No patient demographic information was extracted. Data were exported in CSV format, and tables and figures were generated using Microsoft Excel 2016 (Microsoft, Redmond, WA, USA) with custom macros. These variables were also used to summarize annual examination characteristics, including total examination volume, proportion of contrast-enhanced examinations, and patient type (inpatient vs. outpatient). Figure 2 shows representative visualizations used for systematic assessment of workflow dynamics.

All timestamp variables (reception time, examination start time, and examination end time) were system-generated by the RIS when staff executed the corresponding workflow operations in real time and were not manually entered by staff. Specifically, examination start time was recorded when the RIS examination-start operation was executed, and examination end time was recorded when the RIS examination-end command was executed at the end of the examination. Staff did not manually type timestamp values or enter them retrospectively. In the three-scanner environment, patient entry/exit time for each scanner was calculated as the interval from the preceding patient’s examination end to the current patient’s examination start within the same CT room. Therefore, the first examination of each day on each scanner was excluded from this calculation. No records with missing timestamps were identified in the analyzed dataset. Long idle gaps between examinations were retained if the timestamp sequence was valid, because such intervals represent operational idle time relevant to scanner throughput. No additional duration-based trimming rule or statistical outlier exclusion threshold was applied. Instead, day-level medians were used to reduce the influence of unusually long or atypical per-examination intervals.

### Goal setting, issue identification, and KPI definition

Our CT section often had difficulty completing examinations within regular working hours, resulting in overtime. Accordingly, within this framework, we defined the key goal indicator (KGI) as the end time of the final CT examination, serving as a proxy for completion of daily operations within regular working hours while maintaining the existing examination volume.

**Fig. 3:**
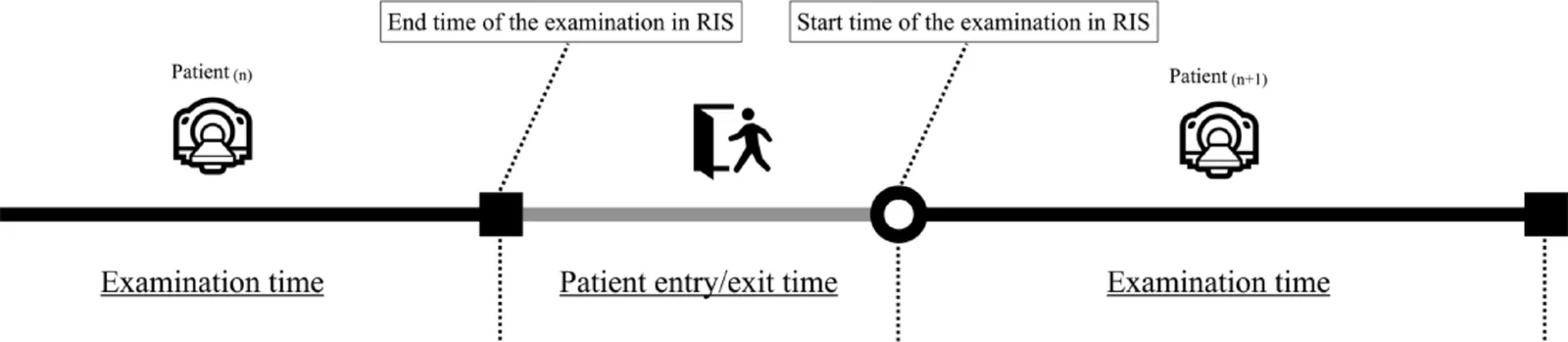
Conceptual framework of the key success factors (KSFs) and key performance indicators (KPIs) derived from consecutive CT examination timelines. The timeline of two consecutive CT examinations was decomposed to define two KPIs. “Examination time” was defined as the interval between the recorded start and end timestamps of an examination and reflects scan-room operational efficiency, including patient positioning, scanning, and immediate post-processing tasks. “Patient entry/exit time” was defined as the interval between the end timestamp of one examination and the start timestamp of the next examination, capturing turnover-related workflow steps around the scan room, such as patient guidance, room turnover, preparation procedures, and patient movement. These intervals were selected because they represent direct, quantifiable determinants of CT throughput. By anchoring the KPIs to discrete timestamps recorded in the RIS, this framework reduces observer-dependent measurement variability and supports reproducible evaluation of workflow performance. This decomposition also distinguishes scan-room efficiency from section-level throughput, providing a basis for interpreting how different types of interventions relate to the observed changes in KPIs and KGI

To identify potential sources of waste and variation, we decomposed the CT service process into six operational stages (reception, waiting area, patient guidance, IV preparation, scan, and exit) and implemented five targeted workflow interventions as iterative KAIZEN actions. We identified two key success factors (KSFs) and operationalized them as quantifiable KPIs: examination time and patient entry/exit time (Fig. 3). Both KPIs were continuously monitored using RIS-generated daily reports (Fig. 2), providing a consistent basis for subsequent comparative analyses.

### Statistical analysis

The workflow intervention (KAIZEN) was initiated in January 2021. For analyses, the study period was categorized into a baseline period (2018–2019), a COVID-19–affected period (2020), and a KAIZEN intervention period (2021–2022). The COVID-19–affected period was defined as calendar year 2020 and treated as a distinct interruption. We set January 2020 as the start of this period because institutional preparatory measures (including infection control planning and workflow reconfiguration) were initiated around that time in response to the emerging outbreak in East Asia and may have affected scheduling and staffing patterns prior to the first major domestic wave [16]. We acknowledge that the operational impact of COVID-19 may have varied within 2020 and extended into 2021; however, as detailed in the Methods (Overview of our CT section) and Discussion, COVID-19 patients were consistently directed to the dedicated emergency CT system and did not use the three imaging center scanners under study. To further assess robustness, sensitivity analyses that varied the COVID-19–affected period were performed and are reported in the Results and in Supplementary Table 3. These included (i) shifting the COVID start date to April 2020, (ii) adding a third post-pandemic breakpoint in January 2022 to allow for a delayed COVID-end level change, and (iii) excluding all 2020 data and fitting a one-breakpoint (KAIZEN-only) model on the remaining pre- and post-KAIZEN years. A total of 150,540 examinations performed during regular weekday working hours were included. The number of analyzed weekdays was 245 in 2018, 242 in 2019, 243 in 2020, 243 in 2021, and 242 in 2022.

Descriptive statistics summarized annual examination characteristics. Categorical variables (contrast enhancement and patient type) were presented as numbers and percentages, and comparisons across years were performed using the chi-square test.

The KPIs (“examination time” and “patient entry/exit time”) were aggregated at the day level as the daily medians across all examinations performed on each weekday. Daily medians were used to provide a robust summary of skewed per-exam time distributions, minimizing the influence of outliers and atypical workflows. The KGI (“end time of the final CT examination”) was evaluated as one value per weekday and modeled as minutes relative to the daytime service end time (17:15; 0 = 17:15), with negative values indicating completion before 17:15.

Interrupted time-series (ITS) analyses were conducted for each outcome using segmented regression with two interruption points: January 2020 (start of the COVID-19–affected period) and January 2021 (start of the KAIZEN intervention). Models included terms for baseline level and slope, immediate level changes at each interruption, and slope changes after each interruption. To account for autocorrelation and heteroskedasticity, standard errors were estimated using heteroskedasticity- and autocorrelation-consistent (HAC; Newey–West) estimators with a maximum lag of 10 days.

For the KPI outcomes, primary ITS models adjusted for daily examination volume, day of the week, and inpatient proportion. These covariates were selected based on established determinants of CT workflow performance whose variation is independent of the KAIZEN intervention [17]. Unadjusted KPI models were also fitted as sensitivity analyses to assess robustness to covariate inclusion. The KGI was analyzed without covariate adjustment using the segmented ITS model described above, because it was defined as an aggregated day-level operational endpoint rather than a per-examination workflow interval; it was therefore interpreted as supportive evidence of section-level completion time. Two-sided *p*-values < 0.05 were considered statistically significant, and all reported *p*-values are uncorrected for multiple comparisons. In addition, the Bonferroni-adjusted threshold for the two KPI outcomes and the KGI operational endpoint is *α* = 0.017; the KAIZEN-associated immediate level-change estimates (*p* = 0.015, *p* < 0.001, and *p* = 0.011, respectively) remain statistically significant under this stricter threshold. All analyses were performed using Python (version 3.9.6) and the statsmodels library (version 0.14.6). Segmented regression models were fitted using ordinary least squares with HAC standard errors implemented in statsmodels.

The five KAIZEN interventions were introduced sequentially over a concentrated period between January and February 2021 (see Supplementary Table 1 for individual timelines). Although staggered introduction was considered in the analytical design, a single interruption point (January 2021) was modeled for two reasons. First, the intervals between individual interventions were too short (days to weeks) to allow reliable estimation of independent level and slope changes for each component. Second, the interventions were designed as a mutually reinforcing package (later interventions built upon workflow foundations established by earlier ones), making individual component attribution methodologically problematic. The January 2021 interruption therefore represents the initiation of the integrated KAIZEN program rather than any single intervention, and the estimated effects should be interpreted accordingly.

## Results

### Interventions in the operational stages reflecting KSFs

Based on the stage-based assessment of the CT examination process, we identified five factors associated with workflow stagnation and implemented five corresponding interventions. Detailed operational definitions and month-level implementation periods are provided in Supplementary Table 1. Through the iterative continuous improvement (KAIZEN) process, the following five interventions were established to address the predefined KSFs:

1. Consolidation of high-efficiency examinations to improve scan-room throughput.
2. Optimization of IV-line placement by the IV nurse, who formally assumed primary responsibility for contrast-enhanced CT examinations, to reduce preparation-related interruptions around the scan room.
3. Adjustment of appointment schedules to redistribute examinations and mitigate peak-time congestion.
4. Active management of the waiting list at reception based on real-time scanner availability to regulate patient inflow into the waiting area.
5. Introduction of a shoes-on policy for ambulatory patients to eliminate a time-consuming step during patient entry/exit.

Across the study periods, trends in KPIs and KGI were evaluated as described below (Fig. 5).

### Trend in the number of CT examinations

**Fig. 4:**
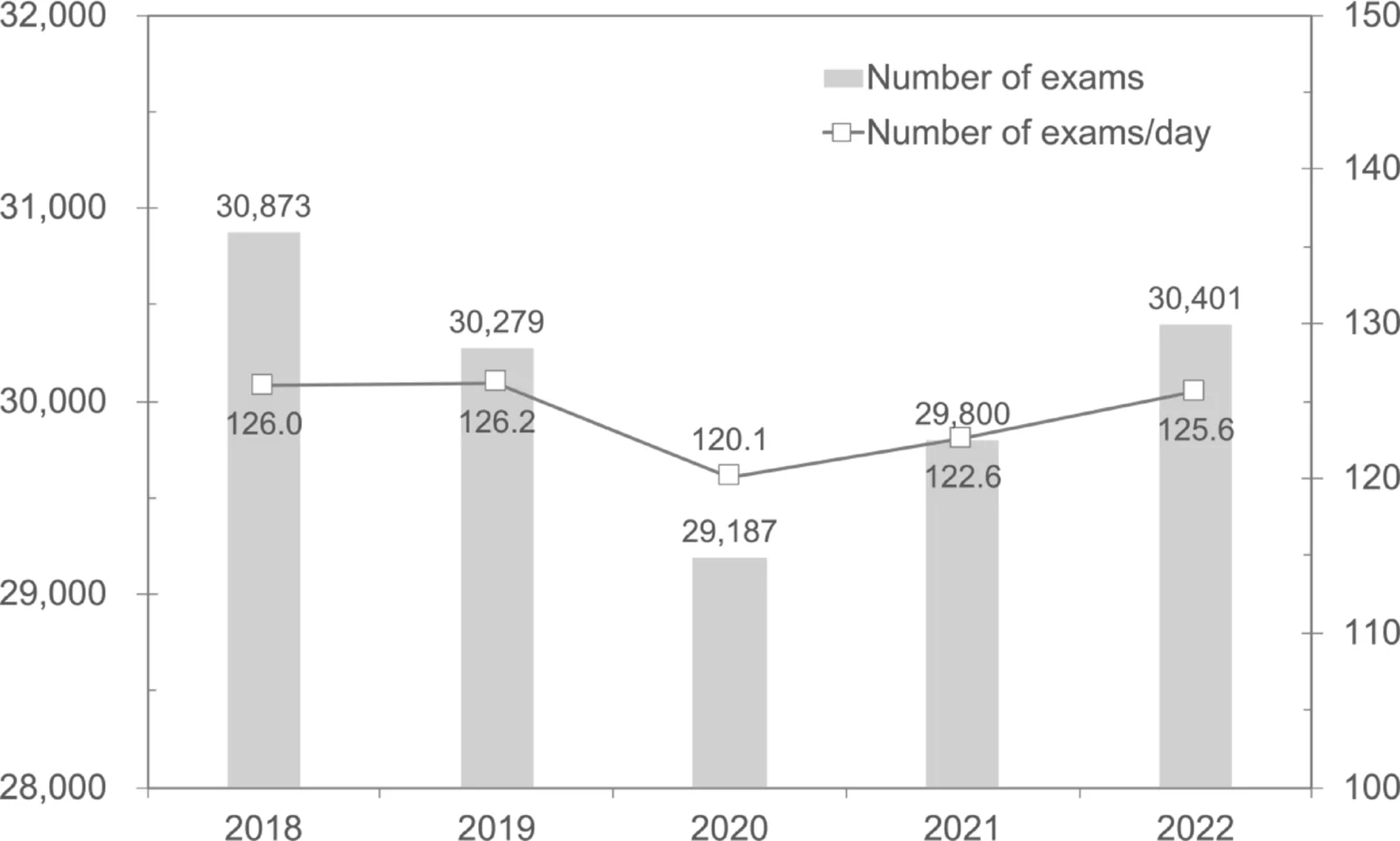
Trend in the number of CT examinations (2018–2022). The bar graph indicates the annual number of CT examinations, and the line indicates the mean number of examinations per weekday. The COVID-19 pandemic caused a reduction in examination volume in 2020 (29,187 examinations; mean 120.1 per weekday), which was treated as a distinct period (COVID-19–affected period) in the segmented regression analysis. Examination volumes recovered in 2021 and 2022, approaching pre-pandemic levels

Annual and daily examination trends from 2018 to 2022 are shown in Fig. 4. Examination volume decreased in 2020 during the COVID-19 pandemic and recovered by 2022. Specifically, the total (30,401) and mean daily (125.6) examinations in 2022 approximated 2018 levels (30,873 and 126.0, respectively). Case composition was broadly similar across years. Although statistically significant differences were observed in the proportions of contrast-enhanced examinations and patient type (*p* < 0.001 for both), variations remained within a narrow range. For example, the proportion of contrast-enhanced examinations ranged from 67.1% to 70.0%, and the inpatient proportion declined from 24.6% (2018) to 20.2% (2022) (Table 1).

**Table 1:** Annual distribution of CT examinations by contrast use and patient type

Year	Total CT examinations	Non-contrast CT, *n* (%)	Contrast-enhanced CT, *n* (%)	Inpatients, *n* (%)	Outpatients, *n* (%)
2018	30,873	10,165 (32.9)	20,708 (67.1)	7,587 (24.6)	23,286 (75.4)
2019	30,279	9,084 (30.0)	21,195 (70.0)	7,030 (23.2)	23,249 (76.8)
2020	29,187	9,243 (31.7)	19,944 (68.3)	6,778 (23.2)	22,409 (76.8)
2021	29,800	9,092 (30.5)	20,708 (69.5)	6,540 (21.9)	23,260 (78.1)
2022	30,401	9,362 (30.8)	21,039 (69.2)	6,126 (20.2)	24,275 (79.8)

Values are shown as number of examinations with percentages in parentheses (percentages are calculated using the annual total as the denominator). Examinations performed during regular weekday working hours are included. Data from 2020 are presented as the COVID-19–affected period. Categorical variables were compared using the chi-square test. Statistically significant differences were observed in the proportions of contrast-enhanced examinations and patient type across years (*p* < 0.001 for both)

### Examination time

**Fig. 5:**
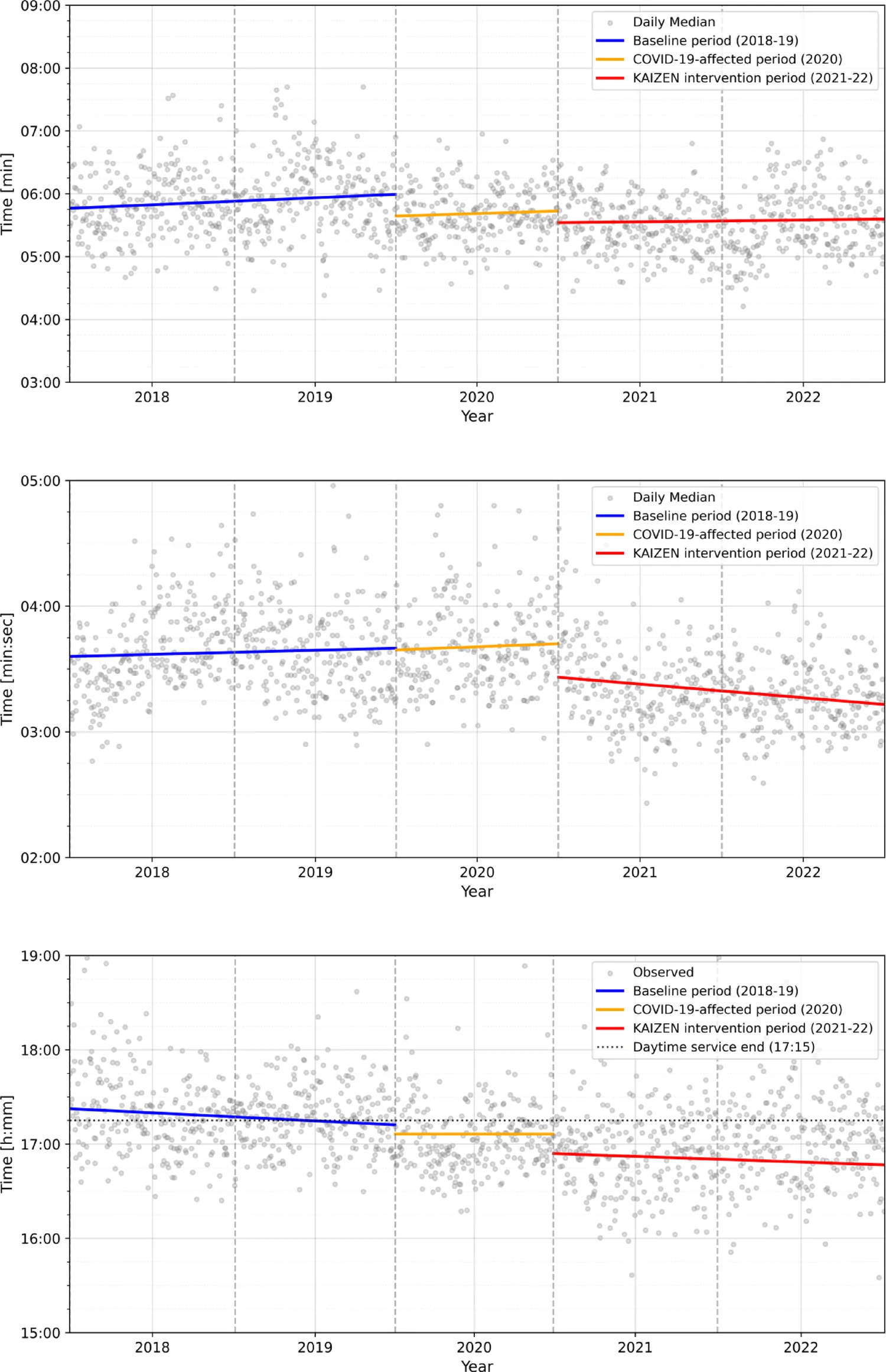
Interrupted time-series (ITS) analyses of key performance indicators (KPIs) and the key goal indicator (KGI) across study periods. **a** ITS analysis of the KPI “Examination time.” Each dot represents the daily median examination time (median duration of CT examinations performed on that weekday). Colored line segments show fitted values from a segmented regression model for the baseline period (2018–2019), the COVID-19–affected period (2020), and the KAIZEN intervention period (2021–2022). Vertical dashed lines indicate annual boundaries and the interruption points (January 2020 and January 2021). Time is shown in mm:ss. **b** ITS analysis of the KPI “Patient entry/exit time.” Dots represent the daily median entry/exit time, and segmented regression fits are shown as in panel (a) for the same three periods. Vertical dashed lines indicate annual boundaries and interruption points (January 2020 and January 2021). Time is shown in mm:ss. **c** ITS analysis of the KGI “End time of the final CT examination,” defined as the end time of the last CT examination on each weekday. Markers represent observed daily end times, and line segments represent fitted values from the segmented regression model for the three periods defined above. Vertical dashed lines indicate annual boundaries and interruption points (January 2020 and January 2021). The horizontal reference line indicates the end of daytime service hours (17:15)

Figure 5a shows the ITS analysis of daily median examination time. In the primary covariate-adjusted segmented regression model with two interruptions (January 2020 and January 2021), the COVID-19–affected period (January 2020) was associated with a significant immediate level decrease of 40.49 s (adjusted *β* =  − 40.49 s; 95% CI, − 72.50 to − 8.52 s; *p* = 0.013), without a significant post-intervention slope change (adjusted *β* = 0.054 s/day; *p* = 0.393). Initiation of KAIZEN in January 2021 was associated with a statistically significant additional immediate level decrease of 17.37 s (adjusted *β* =  − 17.37 s; 95% CI, − 31.35 to − 3.40 s; *p* = 0.015), without a significant post-intervention slope change (adjusted *β* =  − 0.024 s/day; *p* = 0.634).

In the unadjusted sensitivity analysis, the COVID-19–affected period was associated with an immediate level decrease of 20.76 s (*β* =  − 20.76 s; 95% CI, − 34.05 to − 7.47 s; *p* = 0.002), without a significant slope change (*β* =  − 0.006 s/day; *p* = 0.816). The immediate reduction at KAIZEN initiation was directionally consistent but did not reach statistical significance (*β* =  − 11.07 s; 95% CI, − 22.60 to 0.47 s; *p* = 0.060), with no significant post-intervention slope change (*β* =  − 0.012 s/day; *p* = 0.699).

To assess the robustness of these findings to the definition of the COVID-19–affected period, we conducted a sensitivity analysis treating April 2020 (the start of the first national state of emergency in Japan) as the COVID interruption point. In the covariate-adjusted model with this alternative specification, KAIZEN initiation in January 2021 was associated with a statistically significant immediate reduction in examination time (adjusted *β* =  − 27.83 s; 95% CI, − 45.97 to − 9.69 s; *p* = 0.003), consistent with and larger in magnitude than the primary analysis. The unadjusted model with the April 2020 breakpoint showed a directionally consistent but non-significant reduction at KAIZEN initiation (− 10.44 s; 95% CI, − 22.80 to 1.92 s; *p* = 0.098). These findings indicate that the estimated KAIZEN effect on examination time is not sensitive to the precise delineation of the COVID-19–affected period.

To further evaluate whether COVID-19 effects that persisted beyond December 2020 might confound the KAIZEN estimate, we fitted two additional models. In a three-breakpoint specification that added a post-pandemic breakpoint in January 2022, the KAIZEN effect on patient entry/exit time remained significant (adjusted *β* =  − 13.19 s; 95% CI, − 23.88 to − 2.50 s; *p* = 0.016), and the effect on examination time was directionally consistent but non-significant (adjusted *β* =  − 21.58 s; 95% CI, − 47.72 to + 4.55 s; *p* = 0.105), with the reduction in statistical precision reflecting the additional parameters introduced by the third breakpoint. Notably, the estimated post-pandemic level change for January 2022 was itself non-significant for both KPIs (p ≥ 0.29); no discrete COVID-related reversion was observed at this hypothesized recovery point. In a complementary exclusion analysis in which all 2020 data were omitted and a one-breakpoint KAIZEN-only model was fitted to the 2018–2019 and 2021–2022 data, the KAIZEN effect was larger in magnitude and highly significant for both outcomes (examination time: adjusted *β* =  − 36.07 s; 95% CI, − 52.03 to − 20.11 s; *p* < 0.001; patient entry/exit time: adjusted *β* =  − 15.27 s; 95% CI, − 23.94 to − 6.59 s; *p* < 0.001). All sensitivity specifications are tabulated in Supplementary Table 3. An additional three-breakpoint model with a COVID-end breakpoint in April 2021 was evaluated but is not reported because the three-month proximity to the KAIZEN breakpoint produced severe multicollinearity that prevented reliable separation of the two level-change coefficients.

### Patient entry/exit time

Figure 5b presents the ITS analysis of daily median entry/exit time. In the primary covariate-adjusted model, KAIZEN initiation in January 2021 was associated with an immediate level decrease of 15.99 s (adjusted *β* =  − 15.99 s; 95% CI, − 23.93 to − 8.04 s; *p* < 0.001). The primary adjusted model was directionally concordant with the unadjusted sensitivity analysis; the principal finding for this KPI was an immediate reduction at KAIZEN initiation rather than a gradual change over time. The unadjusted sensitivity analysis showed a consistent immediate decrease of 16.02 s (*β* =  − 16.02 s; 95% CI, − 24.36 to − 7.62 s; *p* < 0.001). In the unadjusted model, the post-intervention slope showed a downward trend (*β* =  − 0.036 s/day), although this change was not statistically significant (*p* = 0.156). In the primary adjusted model, neither the immediate level change at the January 2020 interruption (adjusted *β* =  − 2.55 s; 95% CI, − 10.79 to 5.69 s; *p* = 0.544) nor the slope change after that interruption (adjusted *β* = 0.008 s/day; *p* = 0.687) reached statistical significance. Adjusted ITS coefficients for the two KPI models are provided in Supplementary Table 2.

### End time of the final CT examination

Figure 5c presents the ITS analysis of the KGI (end time of the final CT examination) using the day-level segmented model. This analysis was not covariate-adjusted and was interpreted as a supportive aggregate operational endpoint. KAIZEN initiation in January 2021 was associated with an immediate level decrease of 12.49 min (earlier completion relative to 17:15) (*β* =  − 12.49 min; 95% CI, − 22.11 to − 2.87 min; *p* = 0.011). No significant post-intervention slope change was observed (*β* =  − 0.014 min/day; 95% CI, − 0.09 to 0.06; *p* = 0.704).

Neither the immediate level change at the January 2020 interruption (*β* =  − 5.80 min; 95% CI, − 19.55 to 7.95; *p* = 0.408) nor the slope change after that interruption (*β* = 0.021 min/day; 95% CI, − 0.06 to 0.10; *p* = 0.598) was significant.

## Discussion

Improvements in the KPIs and KGI were observed during the KAIZEN intervention period and were temporally associated with improved CT operational performance. Although the concurrent implementation of multiple interventions precludes isolation of individual effects, the time-series analyses help identify where within the workflow the most substantial changes likely occurred.

For KPI outcomes, covariate-adjusted primary analyses confirmed that these modest shifts did not account for the observed reduction in patient entry/exit time. Nevertheless, unmeasured differences in patient complexity and workflow context may still have influenced the results.

The potential influence of COVID-19 on the KAIZEN-period estimates warrants specific discussion. First, the two-interruption ITS model explicitly accounts for the COVID-19–affected period (2020) as a distinct segment, thereby separating COVID-related changes in workflow from KAIZEN-initiated changes. Second, and critically, patients with suspected or confirmed COVID-19 infection were directed to the dedicated emergency CT system throughout the study period and were not scanned using the imaging center CT scanners (see Methods). Consequently, COVID-19-specific infection control protocols (such as extended room ventilation intervals or additional personal protective equipment requirements) were not implemented in the imaging center workflow. Volume recovery after 2020 was addressed through covariate adjustment in the primary analysis and did not substantively alter the direction or significance of estimates. Together, these design features and institutional characteristics reduce the likelihood that residual COVID-19 effects materially confounded the KAIZEN-period results. Additional sensitivity analyses varying both the start and end dates of the COVID-19–affected period (Supplementary Table 3) produced KAIZEN estimates that were directionally consistent with the primary analysis; when all 2020 data were excluded, both KPI estimates were larger in magnitude and statistically significant. A three-breakpoint specification with the COVID-end breakpoint placed in April 2021 was evaluated but produced unstable coefficient estimates owing to collinearity with the January 2021 KAIZEN breakpoint and was therefore not reported.

The ITS analyses provide a temporal perspective on how performance changed. For the KGI, initiation of KAIZEN in January 2021 was associated with an immediate level decrease in the end time of the final CT examination (i.e., earlier completion of weekday operations), without evidence of a sustained slope change. This pattern is consistent with interventions aimed at smoothing daily demand and preventing late-day spillover (e.g., appointment redistribution and waiting list management), which were intended to reduce congestion rather than produce gradual improvement. Accordingly, the KGI should be interpreted as an aggregated operational impact measure reflecting the combined downstream consequences of changes in examination time, patient entry/exit time, and daily demand, rather than as an independent mechanistic endpoint. Because the KGI model was not covariate-adjusted, it provides supportive section-level evidence and should not be interpreted in the same way as the adjusted KPI analyses.

For the KPIs, examination time showed a reduction at the onset of the COVID-19–affected period, whereas the additional immediate decrease at KAIZEN initiation was smaller. The adjusted examination-time model also suggested a modest upward pre-COVID baseline slope (0.038 s/day; *p* = 0.070; Supplementary Table 2^2^; the KAIZEN-associated level decrease therefore occurred against a background of gradual worsening rather than an already improving trajectory. In contrast, patient entry/exit time demonstrated a clear immediate reduction at KAIZEN initiation, without a significant post-intervention slope change. This temporal pattern coincided with implementation of the shoes-on policy for ambulatory patients targeting the entry/exit interface. Although attribution to a specific component remains speculative due to concurrent interventions, the largest immediate change at KAIZEN initiation was observed in patient entry/exit time; scan-room and interface processes may have evolved at different points during the study period.

The adjusted model was designated as the primary KPI analysis because daily examination volume, day of the week, and inpatient proportion are established determinants of CT workflow performance whose variation is independent of the KAIZEN program; failure to account for these factors may obscure true intervention effects. The convergent direction of both analyses supports the inference that KAIZEN initiation was associated with a genuine reduction in examination time.

Although absolute per-examination changes were modest, small reductions in median workflow times can accumulate across high examination volumes, potentially reducing overtime beyond regular service hours and supporting more predictable completion. Because month-level seasonality and holiday-related fluctuations were not modeled explicitly, some residual temporal variation may remain, and their contribution to the observed changes cannot be fully excluded. However, the directional consistency of the primary and sensitivity analyses suggests that seasonality alone is unlikely to explain the KAIZEN-associated findings.

Our approach builds on prior work on radiology workflow improvement efforts. For example, Flug et al. applied KAIZEN principles to improve turnaround times [15]; however, such initiatives can be resource-intensive in terms of preparation, training, and maintenance, which may affect sustainability and staff engagement. In contrast, the present approach utilized routinely collected RIS action logs to support iterative, data-informed adjustments to daily operations. Log-based monitoring provides a practical mechanism for continuous feedback and refinement of CT workflows in routine clinical practice.

Consistent with established quality improvement principles emphasizing measurement and remeasurement [17], we defined KPIs as measurable process indicators associated with the KGI, defined as timely completion of daily CT services. We selected KPIs intended to be meaningful, scientifically sound, and interpretable [18, 19], and RIS action logs offer a practical data source for repeated measurement with minimal additional workload.

This study focused on process and outcome measures derived from RIS action logs and did not explicitly evaluate structural determinants of workflow performance. Although frameworks such as Donabedian’s structure–process–outcome model describe the potential influences of organizational structure, these were beyond the present scope. Because organizational structure, staffing, and institutional culture may influence sustainability of improvement efforts, future multi-center studies should examine how these factors interact with log-driven workflow interventions. Generalizability of these findings is limited by the single-center design and the resource-intensive environment of a large academic hospital with three dedicated CT scanners, an IV nurse, and an on-site radiologist. Institutions with different resource configurations, patient volumes, or information systems may not achieve similar outcomes, and independent replication in diverse settings is needed before broader applicability can be assumed [20, 21].

### Limitations

This study has several limitations. First, as a single-center retrospective before-and-after quality improvement study implementing multiple concurrent interventions, individual component effects cannot be isolated, and residual confounding remains possible. Second, RIS action logs lacked patient-level covariates, limiting adjustment for case complexity. In addition, per-patient implementation fidelity could not be reconstructed retrospectively for any of the five interventions. For the shoes-on policy, we provide an indirect process indicator of sustained post-policy workflow change based on the aggregate entry/exit time distribution (Supplementary Table 4); for the remaining interventions, no comparable retrospective indicator is available because RIS action logs do not record the relevant operational details (e.g., physical location of IV-line placement, scheduled vs. actual time-slot assignment, and reception-level routing decisions). Third, although KPI outcomes were evaluated using covariate-adjusted primary ITS models, residual confounding may not have been fully captured; unadjusted sensitivity analyses produced directionally consistent results for these outcomes. The KGI analysis was not adjusted for daily volume, day of the week, or inpatient proportion, and should therefore be interpreted as supportive aggregate evidence of earlier section-level completion rather than as covariate-adjusted inference. Fourth, outcomes were limited to time-based process measures and did not assess patient outcomes, safety events, or cost-effectiveness. Observed changes may partly reflect documentation practices or monitoring-related behavior change (i.e., a Hawthorne effect) [22, 23], in addition to true workflow improvements. Fifth, analyses were restricted to regular weekday daytime operations; after-hours emergency CT was not evaluated. Sixth, staffing data (including year-by-year counts of CT-assigned radiological technologists, mean years of CT experience, and nurse assignments) were not retrievable in a form suitable for retrospective analysis, and we are therefore unable to characterize quantitative staffing trends across the study period. Consequently, we cannot formally exclude the possibility that gradual changes in team composition, staff skill levels, or informal learning effects contributed to the observed improvements. This limitation is particularly relevant to interventions such as “consolidation of high-efficiency examinations” and “appointment schedule adjustment,” whose outcomes depend substantially on staff judgment and experience. Future prospective studies should document staffing profiles alongside workflow metrics. Multi-center studies with standardized log definitions and component-separated designs are warranted.

## Conclusion

In this single-center retrospective before-and-after quality improvement study, RIS action logs captured temporal changes during a KAIZEN-based workflow improvement approach. Improvements included shorter patient entry/exit time and earlier completion of daily operations at KAIZEN initiation. Examination time was significantly reduced in the primary covariate-adjusted ITS analysis; the unadjusted sensitivity analysis showed a directionally consistent but non-significant result. COVID-19-specific protocols did not apply to the imaging center CT workflow, as COVID-19 patients were redirected to a separate emergency CT system. Although causal attribution and downstream outcomes cannot be confirmed within this design, the results suggest that routinely collected RIS logs can support performance monitoring and continuous quality improvement in CT operations. Replication in diverse institutional settings is warranted to assess the generalizability of this approach.

## Supplementary Information

Below is the link to the electronic supplementary material.

Supplementary Material 1

Supplementary Material 2

Supplementary Material 3

Supplementary Material 4
